# Mitochondrial dysfunction in sepsis is associated with diminished intramitochondrial TFAM despite its increased cellular expression

**DOI:** 10.1038/s41598-020-78195-4

**Published:** 2020-12-03

**Authors:** Tim Rahmel, Britta Marko, Hartmuth Nowak, Lars Bergmann, Patrick Thon, Katharina Rump, Sebastian Kreimendahl, Joachim Rassow, Jürgen Peters, Mervyn Singer, Michael Adamzik, Björn Koos

**Affiliations:** 1 Klinik für Anästhesiologie, Intensivmedizin und Schmerztherapie, Universitätsklinikum Knappschaftskrankenhaus Bochum, In der Schornau 23-25, 44892 Bochum, Germany; 2 Institut für Biochemie und Pathobiochemie, Abteilung für Zellbiochemie, Ruhr-Universität Bochum, Bochum, Germany; 3 Klinik für Anästhesiologie und Intensivmedizin, Universität Duisburg-Essen & Universitätsklinikum Essen, Essen, Germany; 4 Bloomsbury Institute of Intensive Care Medicine, Division of Medicine, University College London, London, UK

**Keywords:** Sepsis, Sepsis, Infectious diseases, Acute inflammation

## Abstract

Sepsis is characterized by a dysregulated immune response, metabolic derangements and bioenergetic failure. These alterations are closely associated with a profound and persisting mitochondrial dysfunction. This however occurs despite increased expression of the nuclear-encoded transcription factor A (TFAM) that normally supports mitochondrial biogenesis and functional recovery. Since this paradox may relate to an altered intracellular distribution of TFAM in sepsis, we tested the hypothesis that enhanced extramitochondrial TFAM expression does not translate into increased intramitochondrial TFAM abundance. Accordingly, we prospectively analyzed PBMCs both from septic patients (n = 10) and lipopolysaccharide stimulated PBMCs from healthy volunteers (n = 20). Extramitochondrial TFAM protein expression in sepsis patients was 1.8-fold greater compared to controls (p = 0.001), whereas intramitochondrial TFAM abundance was approximate 80% less (p < 0.001). This was accompanied by lower mitochondrial DNA copy numbers (p < 0.001), mtND1 expression (p < 0.001) and cellular ATP content (p < 0.001) in sepsis patients. These findings were mirrored in lipopolysaccharide stimulated PBMCs taken from healthy volunteers. Furthermore, TFAM-TFB2M protein interaction within the human mitochondrial core transcription initiation complex, was 74% lower in septic patients (p < 0.001). In conclusion, our findings, which demonstrate a diminished mitochondrial TFAM abundance in sepsis and endotoxemia, may help to explain the paradox of lacking bioenergetic recovery despite enhanced TFAM expression.

## Introduction

Sepsis is defined as an acute organ dysfunction caused by a dysregulated immune response to an infection, affecting millions of individuals per year worldwide and representing a major healthcare concern^1,2^. Interest has increasingly focused on the link between sepsis-associated organ failure and mitochondrial dysfunction^3,4^. Mitochondria generate most of the adenosine triphosphate (ATP) required for normal cellular function, but are also involved in multiple intracellular signaling and regulatory processes such as intracellular calcium regulation and production of reactive oxygen species^5–7^. These important regulatory mechanisms seems to be profoundly disturbed in human sepsis, which can ensue mitochondrial dysfunction and reduced oxidative ATP production^3,4,8^.

Impaired mitochondrial functionality and ability to recover likely contribute to organ dysfunction and death^3,4,9,10^. However, mitochondrial dysfunction seems to be highly variable and should not be seen as general denominator for multiple organ failure in sepsis and septic shock^11^. However, describing potential cellular and mitochondrial abnormalities could help to improve our still insufficient understanding about mitochondrial dysfunction in sepsis. Generally, mitochondrial injury and ATP depletion trigger an increased activation of mitochondrial biogenesis, aimed to ameliorate the cellular effects of mitochondrial dysfunction^12–14^, and involves a signaling network that converges on the nuclear-encoded mitochondrial transcription factor A (TFAM)^12^. TFAM regulates de novo synthesis of mitochondrial proteins, facilitates mitochondrial DNA replication, and mediates mitochondrial DNA protection^15,16^. TFAM is a ~ 24 kDa protein with non-specific DNA-binding properties. After cytosolic synthesis as a precursor protein (~ 29 kDa), TFAM is shuttled to the mitochondria, crossing the outer and inner membranes. Mature TFAM is then generated by cleavage of a targeting sequence (~ 5 kDa) by a processing peptidase in the mitochondrial matrix^17,18^. Lack of mature TFAM entails mitochondrial dysfunction and an energy crisis, with insufficient TFAM resulting in possible death^19^. The ability to resolve a critical condition such as sepsis-induced organ failure hence could depend on the ability to increase intramitochondrial TFAM abundance so as to restore adequate mitochondrial function^10,12,17,20–22^. Specifically, TFAM plays a central role in the mitochondrial core transcription initiation complex^23^ that is required not only for expression of mitochondrial-encoded respiratory chain subunits but also for mitochondrial DNA replication^24,25^.

Recent studies, however, provide growing evidence that activation of mitochondrial biogenesis in sepsis, although associated with an increased intracellular TFAM expression, is not necessarily accompanied by recovery of mitochondrial function^10,26–28^. This raises the question as to whether steps in TFAM`s production and activity, from nuclear transcription to intramitochondrial actions, are disturbed. Specifically, the functionally important intramitochondrial TFAM has not been explored in cells from septic patients or cellular surrogate models of sepsis.

Accordingly, to test the hypothesis that enhanced TFAM gene expression does not translate into an increased abundance of intramitochondrial TFAM and maintenance of mitochondrial dysfunction, we studied both blood mononuclear cells (PBMCs) from sepsis patients and lipopolysaccharide (LPS)-stimulated PBMCs drawn from healthy volunteers.

## Results

Baseline characteristics of the septic patients are shown in Table 1. The SOFA score at inclusion was 10 ± 4 and 9 patients required norepinephrine for blood pressure support. Thirty-day mortality was 40%. The 20 healthy volunteers consisted of 9 females and 11 males with a mean age of 39 years ± 9. The age-adapted subgroup of controls (n = 9) consisted of 4 females and 5 males with a mean age of 54 years ± 7.

**Table 1:** Baseline characteristics of sepsis patients.

Variable	Sepsis patients (n = 10)
Age [years], mean (± SD)	58 ± 13
Male sex	6 (60%)
Body mass index [kg/m^2^]	27.1 ± 3.8
**Site of infection**	
Pneumonia	5 (50%)
Abdominal infection	3 (30%)
Skin and soft tissue infection	1 (10%)
Urinary tract infection	1 (10%)
**Culture results**	
Gram positive isolates only	3 (30%)
Gram negative isolates only	4 (40%)
Mixed bacterial isolates	1 (10%)
Negative cultures	2 (20%)
C-reactive protein concentration [mg/dL]	22.9 ± 11.5
Procalcitonin concentration [ng/mL]	12.1 ± 24.2
**Leukocyte concentration [10**^**9**^**/L]**	17.5 ± 7.9
Neutrophils [%]	82.9 ± 5.3
Eosinophils [%]	1.6 ± 2.6
Basophils [%]	0.3 ± 0.2
Lymphocytes [%]	8.3 ± 4.2
Monocytes [%]	6.9 ± 4.0
Simplified Acute Physiology Score	43 ± 18
Sepsis-related Organ Failure Assessment score	10 ± 4
Continuous hemofiltration/dialysis	6 (60%)
Mechanical ventilation	5 (50%)
Serum lactate concentration [mg/dL]	1.8 ± 1.0
Norepinephrine therapy	9 (90%)
Death within 30 days	4 (40%)

Data are presented as n (%) or mean (± SD), as appropriate. The presented characteristics refer to baseline measurements recorded on study inclusion. There were no missing data.

**Figure 1:**
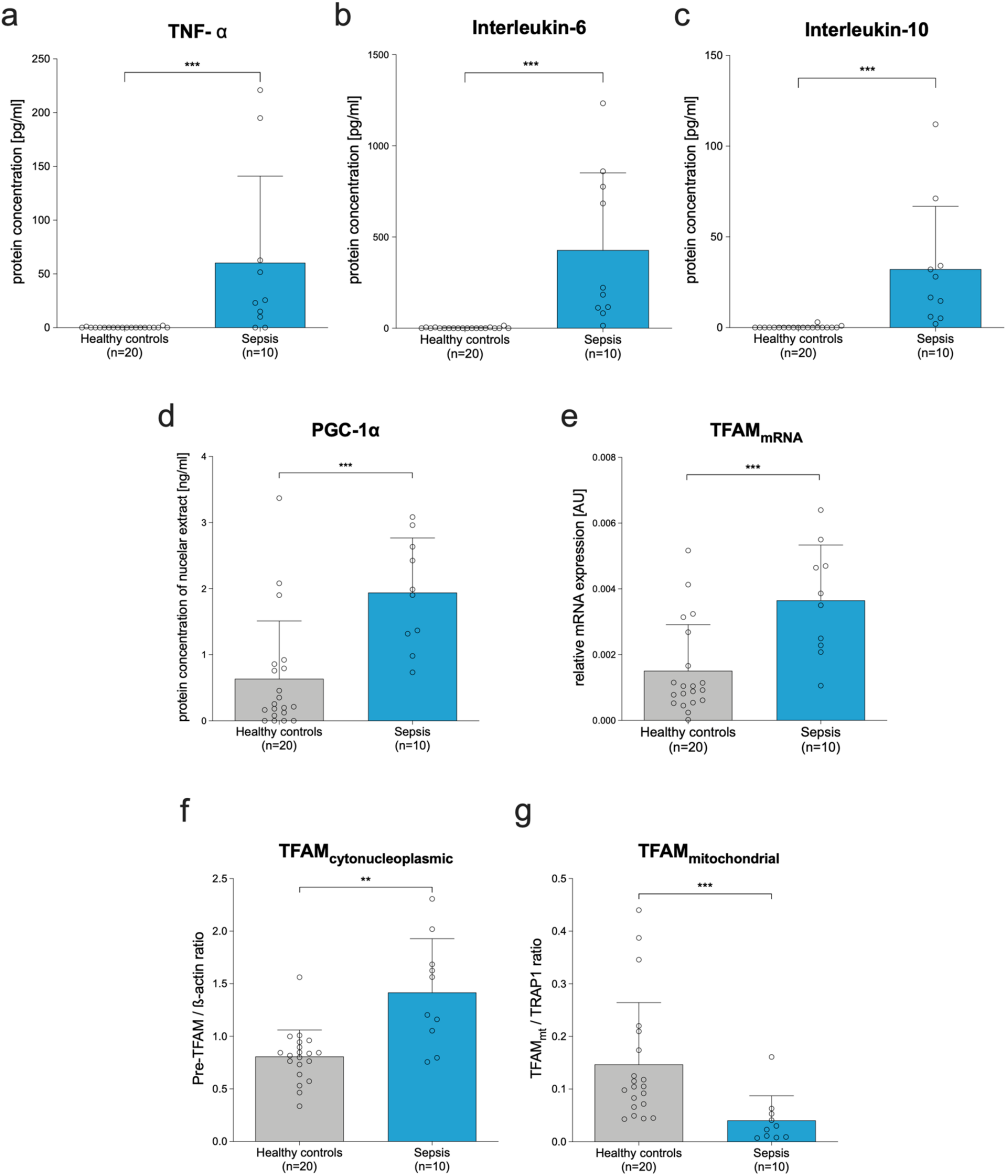
Sepsis is associated with increased extramitochondrial but diminished intramitochondrial TFAM abundance. Results representing PBMCs from sepsis patients (n = 10; blue bars) sampled within 24 h after the diagnosis of sepsis compared to healthy controls (n = 20; grey bars). Upper panel: Concentration of selected cytokines serum of septic patients and healthy controls. (**a**) TNF-α, (**b**) Interleukin-6, and (**c**) Interleukin-10. (**d**) Peroxisome proliferator-activated receptor gamma coactivator 1-alpha (PGC-1α) level (ELISA of nuclear protein extracts). (**e**) Relative TFAM mRNA expression (quantitative polymerase chain reaction; compared to beta actin) of PBMCs; AU: arbitrary units. (**f**,**g**) Relative TFAM protein expression in cytonucleoplasm normalized to beta actin (**f**) and relative intramitochondrial TFAM protein amount normalized to TNF receptor-associated protein 1 (**g**). Each circle represents an individual volunteer/patient; columns with error bars represent mean and SD. There were no missing data. P-values were determined using the Mann–Whitney test; *p < 0.05, **p < 0.01, ***p < 0.001. Cytokine concentrations pg/mL were derived from a calibration curve. All exact values are presented in the Source Data file.

Serum of septic patients demonstrated manifold greater concentrations of TNF-α (60 pg/mL ± 80; p < 0.001; Fig. 1a); interleukin-6 (428 pg/mL ± 423; p < 0.001; Fig. 1b), and interleukin-10 (35 pg/mL ± 11; p < 0.001; Fig. 1c) compared to serum of healthy controls (0 pg/mL ± 1; 2 pg/mL ± 4; 0 pg/mL ± 1, respectively). This was accompanied by an approximately threefold increase in nuclear PGC-1α protein concentration (Fig. 1d; p < 0.001) and of TFAM mRNA expression (Fig. 1e; p < 0.001) in the PBMCs, indicating an activated mitochondrial biogenesis. In this context, we also found a 1.8-fold greater extramitochondrial TFAM protein expression in sepsis patients compared to healthy controls (p = 0.001; Fig. 1f). However, the functionally important intramitochondrial TFAM abundance was approximate 80% less than in controls (p < 0.001; Fig. 1g).

**Figure 2:**
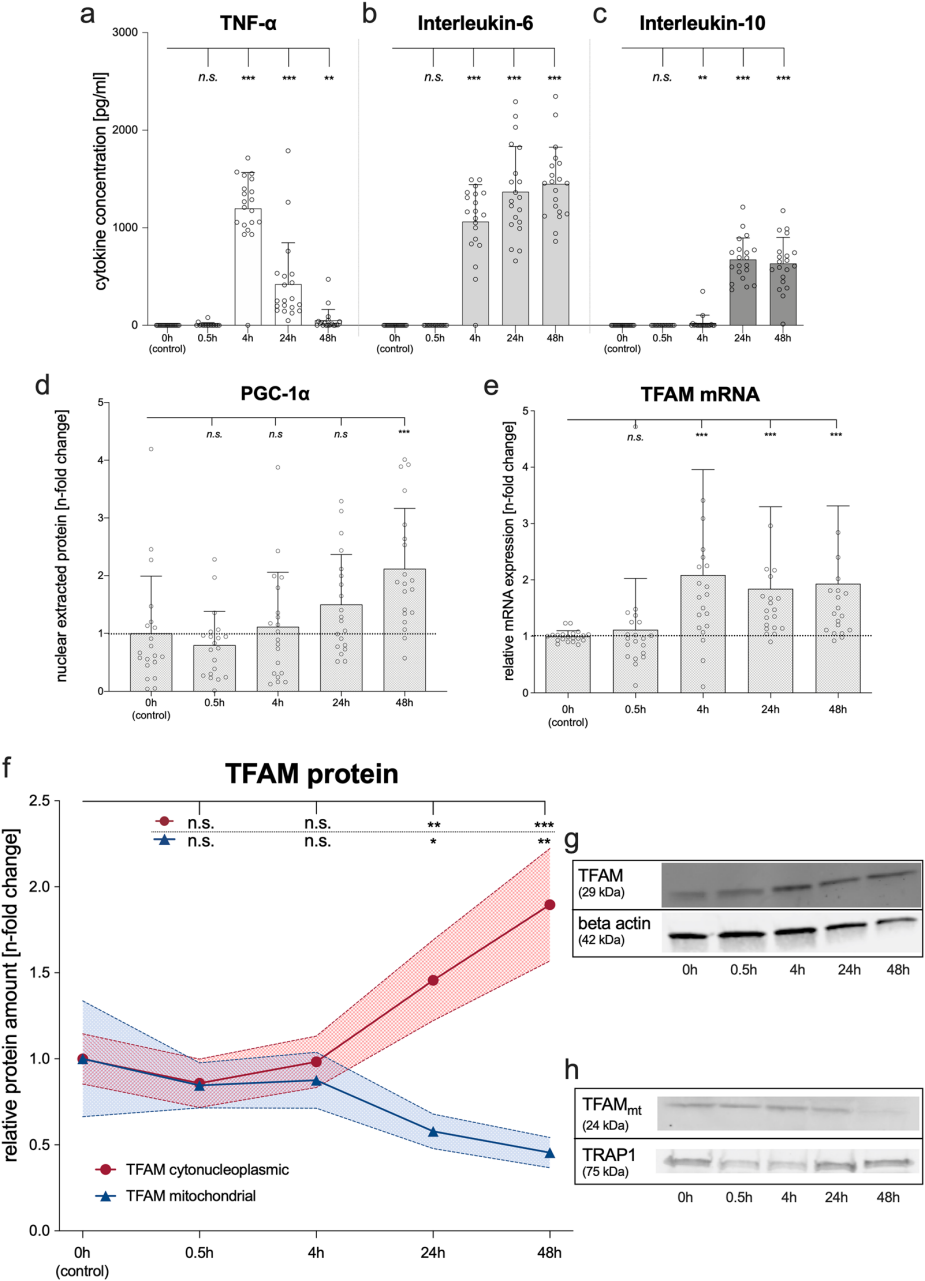
Lipopolysaccharide (LPS) increases extramitochondrial but not intramitochondrial TFAM abundance. LPS stimulation of PBMCs from healthy volunteers before and after 0.5, 4, 24, and 48 h, respectively. Upper panel: Concentrations of selected cytokines in PBMC cell culture supernatants. (**a**) TNF-α, (**b**) Interleukin-6, (**c**) Interleukin-10. (**d**) PGC-1α (ELISA of nuclear protein extracts) in PBMCs of healthy volunteers. (**e**) TFAM mRNA expression (quantitative polymerase chain reaction) normalized to beta actin in LPS-stimulated PBMCs of healthy volunteers (n-fold change to control). (**f**–**h**) TFAM protein expression in LPS-stimulated PBMCs from healthy volunteers. The time course of TFAM protein expression in cytonucleoplasm was normalized to beta actin (red) and the relative intramitochondrial TFAM protein amount was normalized to TNF receptor-associated protein 1 (blue). Values shown are means with corresponding 95% CI (hatched area) of the n-fold change compared to unstimulated PBMCs at baseline. (**g**,**h**) Representative Western Blot images. Full images are provided as Supplementary Figures 7 and 8. Each circle represents an individual experiment; columns with error bars represent means with SD. P values relate to Wilcoxon Test; *p < 0.05, **p < 0.01, ***p < 0.001, and *ns* designates no statistically significant difference. There were no missing data. Cytokine concentrations were derived from a calibration curve. All exact values are presented in the Source Data file.

All these observations were replicated in LPS-stimulated PBMCs from healthy volunteers serving as controls (Fig. 2a–h). Here, LPS stimulation of PBMCs also evoked a 1.5-fold increase of extramitochondrial TFAM protein at 24 h (p = 0.003) and a twofold increase at 48 h (p < 0.001) compared to unstimulated controls (Fig. 2f,g). However, the functionally important intramitochondrial TFAM diminished over time despite the increase in extramitochondrial TFAM (Fig. 2f,h). Indeed, intramitochondrial TFAM had halved at 24 h (p = 0.038) and further decreased to 40% at 48 h (p = 0.002). Thus, LPS stimulation mirrored the altered intracellular distribution of TFAM showing a decreased intramitochondrial presence despite increased extramitochondrial presence.

**Figure 3:**
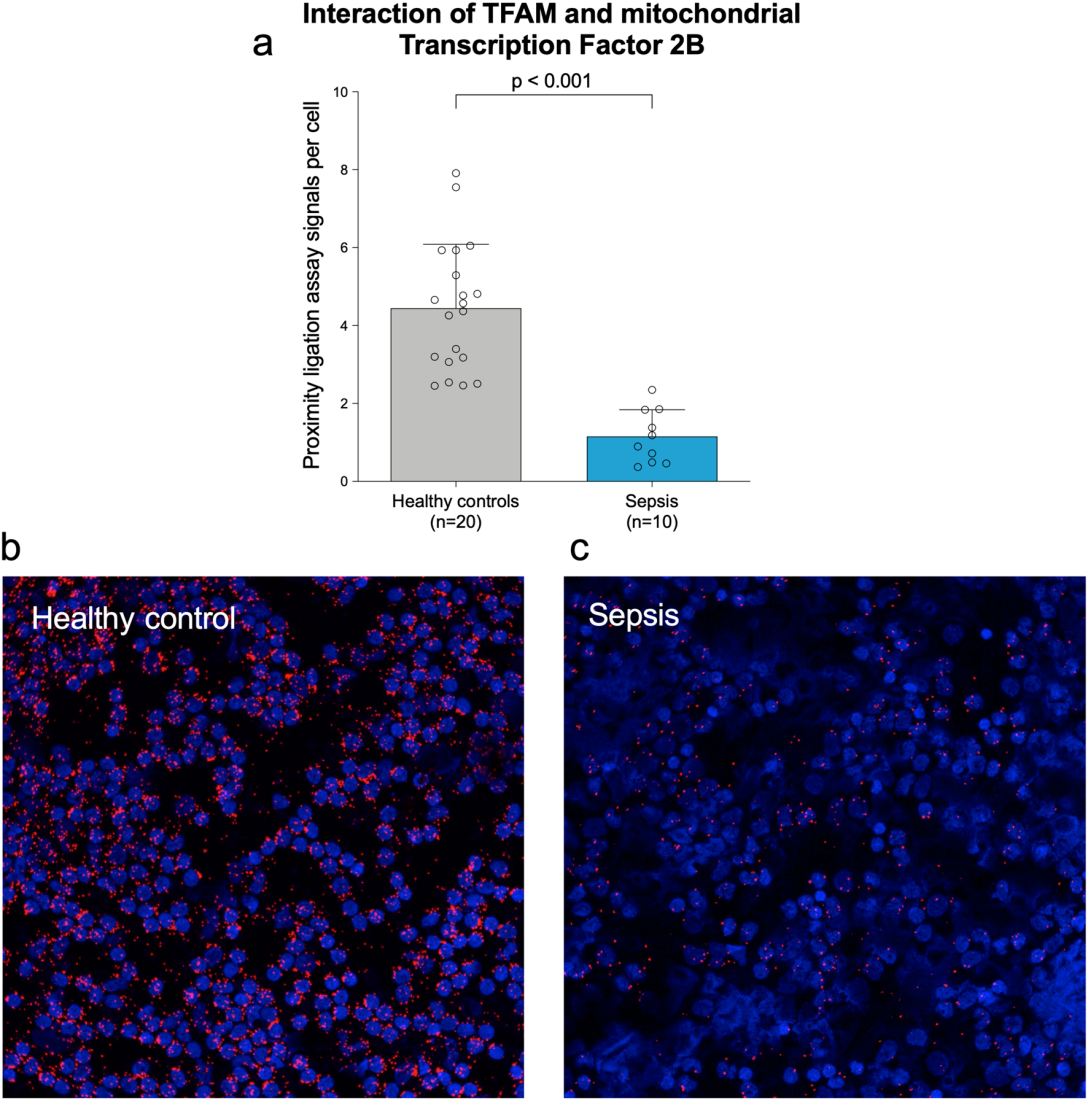
Interaction of TFAM with TFB2M in the human mitochondrial transcription initiation complex is markedly decreased in septic patients. Upper panel: (**a**) Average numbers of signals per cell (mean ± SD) derived from more than 300 analyzed cells for each of the 20 healthy volunteers and 10 septic patients. Each circle represents an individual volunteer/patient. P-values refer to a Mann–Whitney test. There were no missing data. All exact values are presented in the Source Data file. Lower panel: (**b**,**c**) Representative images are shown for a healthy volunteer (**b**) and a sepsis patient (**c**). Red dots reflect protein–protein complex formation; nuclei are counterstained with DAPI (blue).

To assess the functional relevance of intramitochondrial TFAM in PBMCs from sepsis patients, we quantified the interactions of TFAM and mitochondrial Transcription Factor 2B, also known as the mitochondrial core transcription initiation complex (Fig. 3a–c). Here, we found a marked decrease of 74% in PLA signals per cell, as a measure of protein interactions (Fig. 3a, p < 0.001), when comparing PBMCs from septic patients (1.2 signals per cell; 95%-CI 0.7 to 1.6, Fig. 3c) to controls (4.5 signals per cell; 95%-CI 3.7 to 5.2, Fig. 3b), in line with diminished mitochondrial TFAM. Of special clinical interest, the diminished protein interactions of TFAM with TFB2M in PBMCs of septic patients inversely correlated with the SOFA score (r^2^ = 0.58 ; p = 0.011). This may indicate a potential association between intramitochondrial TFAM abundance and severity of the sepsis-related organ dysfunction.

**Figure 4:**
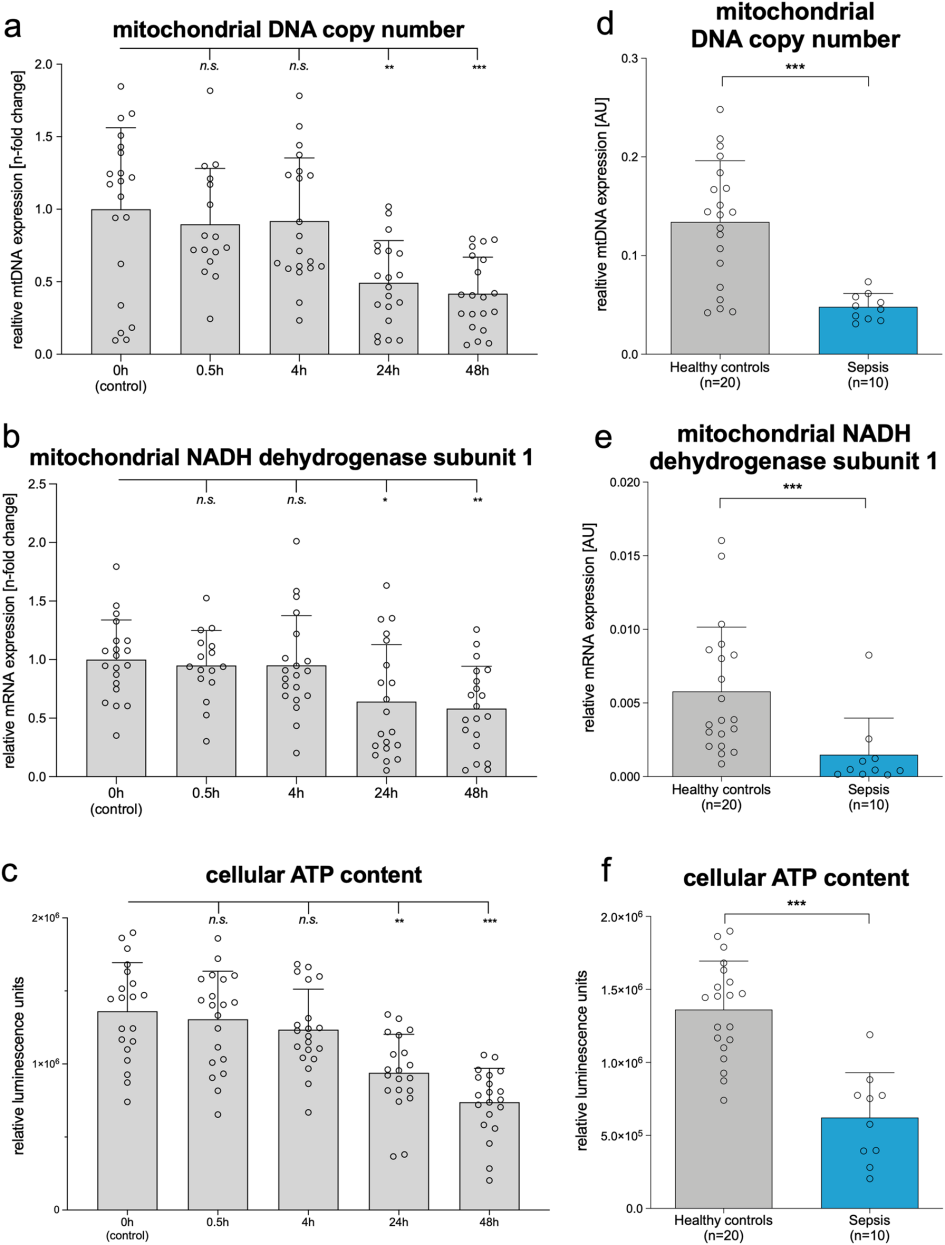
Deterioration of mitochondrial function in LPS-stimulated PBMCs from healthy volunteers’ mirrors findings in sepsis patients. Left panel: Time course of mitochondrial function indicators (**a**) mitochondrial DNA copy number, (**b**) mitochondrial NADH dehydrogenase subunit 1, and (**c**) cellular ATP amount) in PBMCs from healthy volunteers before and 0.5, 4, 24, and 48 after LPS stimulation. Right panel: Mitochondrial function indicators (**d**) mitochondrial DNA copy number, (**e**) mitochondrial NADH dehydrogenase subunit 1, and (**f**) cellular ATP amount) of PBMCs from septic patients (blue bars) sampled within 24 h after onset of sepsis compared to healthy controls (grey bars); AU: arbitrary units. (**a**,**d**) Mitochondrial DNA copy number. (**b**,**e**) mRNA of mitochondrial encoded mitochondrial NADH dehydrogenase subunit 1. (**c**,**f**) Cellular ATP was determined using a luciferase-based assay and expressed as relative fluorescent units normalized to 2.25 × 10^5^ cells per well. (**a**–**f**) Each circle represents an individual volunteer or patient; columns with errors bar represent means with SD. P-values were determined using the Wilcoxon test in (**a**,**b**,**c**) and the Mann–Whitney test in (**d**,**e**,**f**) *p < 0.05, **p < 0.01, ***p < 0.001, and *ns* for no statistically significant difference. There were no missing data. All exact values are presented in the Source Data file.

In parallel with the findings in LPS stimulated PBMCs from healthy volunteer controls (Fig. 4a–c), mitochondrial DNA copy number decreased in septic patients’ PBMCs by almost 70% (Fig. 4d; p < 0.001) compared to healthy controls. Mitochondrial NADH dehydrogenase subunit 1 mRNA decreased by over 80% (Fig. 4e; p < 0.001) and cellular ATP content decreased by 60% (Fig. 4f; p < 0.001).

## Discussion

Our study reveals that TFAM abundance in mitochondria decreases during the early inflammatory phase of sepsis, despite cellular upregulation of TFAM expression. Deprivation of intramitochondrial TFAM in turn was associated with reductions in mitochondrial DNA copy number, mitochondrial NADH dehydrogenase subunit 1 expression, and decreased cellular ATP content, all suggesting decreased cellular energy supply.

Over the last two decades, several studies have provided substantial evidence that sepsis-related organ failure may relate to mitochondrial dysfunction and lack of bioenergetic recovery^3,9,29–32^. Mitochondrial recovery mainly depends on a sufficient upregulation of mitochondrial biogenesis^10,33^. Notably, the early inflammatory response in sepsis amplifies expression and activation of factors stimulating mitochondrial biogenesis and repair such as TFAM, in line with our findings^10,14,34^. Indeed, it seems evolutionarily prudent that an inflammatory response, especially to sepsis, triggers TFAM expression to mitigate harm inflicted by excessive inflammation.

In line with other studies, we found an early upregulation of TFAM mRNA and an increase in extramitochondrial TFAM protein in septic patients that was mirrored within 24 to 48 h by LPS stimulation of PBMC from healthy volunteers^10,26,33,34^. High TFAM concentrations have been described as clinically beneficial, potentially alleviating organ dysfunction whereas a lack of TFAM has detrimental effects on organ function and outcome^12,19,20^. While the correlation between mitochondrial function in PBMCs and that of organs remains contested, investigations of cells from solid organs would require invasive biopsies which would hardly be ethically justified. However, tests of peripheral blood PBMCs have been proposed to offer valid information about “general” mitochondrial health^35^. Thus, the timely (within the first 24 h) increases in cellular TFAM expression in PBMCs of septic patients and in endotoxemia, as supported by our results, may be beneficial and promote mitochondrial recovery. Nevertheless, our data cannot prove a causal correlation or fixed association of mitochondrial function between peripheral blood cells and other solid organs in sepsis, especially since other studies in this context provide heterogeneous results needing further clarification^36–39^.

As shown by the progressive decrease both in mitochondrial DNA copy number and ATP content, mitochondrial function still deteriorated despite increased cellular TFAM concentrations. A compromised mitochondrial biogenesis despite an early activation of important transcription factors has already been described by others, including investigations on mitochondrial function in cardiomyocytes^10,26,32^. Remarkably, following LPS stimulation, impaired recovery of the mitochondrial respiratory chain was observed despite an increase in the nuclear transcription factor PGC-1α, a master regulator of mitochondrial biogenesis^27^. In this context, our results shed light on a potential intracellular maldistribution of TFAM in sepsis and endotoxemia resulting in decreased intramitochondrial TFAM, although its source, i.e., extramitochondrial TFAM, is conserved or even increased.

Nevertheless, there are several questions that need further clarification. For example, whether this problem is TFAM specific or also affects other mitochondrial proteins? Are the observed findings different in cells of solid human organs, in particular brain, kidney, heart, and liver? Furthermore, do our observations in PBMCs from sepsis patients and LPS-stimulated controls reflect an adaptive re-programming of electron transport chain function rather than a primary damage inflicting mechanism of sepsis? Although further exploration of molecular mechanisms potentially responsible for our observations was beyond the scope of the present study these questions warrant further work.

A strength of our study is, that we could independently support our finding of decreased intramitochondrial TFAM by a dramatically diminished TFAM/TFB2M protein interaction rate. This protein interaction makes up the human mitochondrial transcription initiation complex. A decrease of this interaction was accompanied by a profoundly affected mitochondrial transcription and replication machinery, as shown by decreases in mitochondrial DNA copy number, mitochondrial NADH dehydrogenase subunit 1 mRNA expression, and cellular ATP content. Therefore, TFAM seems not to appear at its proper site of action where it is needed. However, our data does not allow to decide whether the described phenomenon is a TFAM-specific problem or whether several proteins are affected simultaneously. Therefore, the results of our PLA could further be influenced by a decreased TFB2M concentration or post transcriptional modifications that are currently unknown.

Though our data do not provide causal or even more detailed mechanistic molecular insights, our findings provide reason to speculate that a diminished intramitochondrial TFAM concentration may impair mitochondrial recovery and energetics. We also hypothesize that it may contribute to organ dysfunction and death from sepsis. In this context, a hampered mitochondrial protein import may represent an interesting mechanism evoking diminution of intramitochondrial TFAM^40,41^. Recent studies also indicate that the complex mitochondrial protein import is strictly regulated, suggesting a remarkable diversity of potentially mechanisms underlying our findings^40^. Other explanations are also possible. For example, a higher proteolytic activity in the mitochondrion in sepsis and endotoxemia could also explain our results.

Our data do not provide a definitively answer as to which mechanisms are responsible for our observations. In addition, a detailed investigation of the mitochondrial oxidative metabolism may have expanded our mechanistic insights but was beyond the scope the present study**.** Therefore, further research is warranted to elucidate the molecular mechanisms underlying the alterations in cellular and intramitochondrial TFAM distribution, its causes, and consequences. Aside from mechanistic considerations, it is intriguing to speculate that the extent and duration of the apparent intracellular TFAM maldistribution might represent a prognostic biomarker, since TFAM/TFB2M protein interactions strongly correlated with the SOFA score of our sepsis patients. Further studies in larger groups of patients are needed to explore this hypothesis.

## Conclusion

Despite increased extramitochondrial TFAM both intramitochondrial TFAM abundance and the functionally important protein interaction with TFB2M are decreased in PBMCs from sepsis patients. This is associated with a decreased mtDNA copy number and cellular ATP content suggesting a mitochondrial dysfunction. All these findings can be replicated when PBMCs are exposed to LPS. Taken together this suggests that intracellular TFAM maldistribution could be an important feature in sepsis. This calls for further studies investigating the molecular mechanisms responsible for the observed findings.

## Materials and methods

### Study design and oversight

We conducted a prospective, observational, single-center, in vitro and in vivo study registered in the German clinical trials database (DRKS00015619) prior to first patient enrollment. The Ethics Committee of the Medical Faculty of the Ruhr-University of Bochum (protocol no. #18-6257) reviewed and approved the study and written informed consent was obtained from healthy subjects and patients or their guardians, as appropriate. This study was conducted in accordance with the revised Declaration of Helsinki, good clinical practice guidelines, and local regulatory requirements.

### Patient and volunteer cohorts and treatments

We recruited twenty healthy subjects from the Medical Faculty of the Ruhr-University Bochum between October 10 and December 21, 2018 who free from infection for at least 4 weeks prior to study participation. Blood was drawn and PBMCs isolated as described below. For subsequent experiments PBMCs of healthy subjects were seeded at a density of 2 × 10^7^ cells per well and incubated with or without 10 µg/mL LPS (Supplementary Figure 1; *Escherichia coli* type 0111:B4; L4391, Sigma-Aldrich, St.Louis, MI). Serial in vitro measurements were performed at baseline prior to LPS stimulation and at 0.5, 4, 24, and 48 h.

Septic patients were considered eligible if they fulfilled the criteria for sepsis as defined by the current Sepsis-3 definition and enrollment, written informed consent and blood sampling had been completed within the first 24 h after diagnosis of sepsis^1^. Exclusion criteria were age under 18 years, pregnancy, pre-existing anemia, known mitochondrial disorder, and the decision to withhold or withdraw life-sustaining treatment on the day of study inclusion. Ten septic patients admitted to the intensive care unit (ICU) of the University Hospital Knappschaftskrankenhaus Bochum between December 3, 2018 and February 28, 2019 were included. PBMCs of these patients were isolated as described below. Cells were not stimulated and directly processed. We followed all patients for 30-day survival commencing from the day of the diagnosis of sepsis.

### Isolation of peripheral blood mononuclear cells

Peripheral blood mononuclear cells (PBMCs) were isolated using a density gradient centrifugation protocol (Ficoll Paque solution, GE Healthcare Bio Science AB, Uppsala Sweden). Briefly, cells were centrifuged in Ficoll Paque solution, forming a PBMC-rich layer that was collected. PBMCs of septic patients were directly processed, as described below. We resuspended isolated cells of healthy volunteers in full RPMI 1640 medium (Invitrogen, Carlsbad, CA) containing 10% fetal calf serum (FCS) (Biochrom AG, Berlin, Germany), 100 U/mL penicillin plus 100 μg/mL streptomycin (both Invitrogen), and held at 37 °C in a humidified atmosphere containing 5% CO_2_ until further use.

### Isolation of mitochondria

To assess and compare intramitochondrial TFAM concentrations to their mitochondria-free cytonucleoplasm, the mitochondria were isolated for each measurement, adapted from the protocol Argan et al. published^42^. Briefly, the supernatant of the PBMCs was collected for quantification of TNF-α, IL-6, and IL-10 as described below. For septic patients’ blood serum was used. The cells were then osmotically swelled and mechanically shredded (homogenized) to release the mitochondria. The mitochondria were then separated from the cytonucleoplasm and cellular debris by different centrifugation steps. Mitochondria were then lysed and protein was isolated. The quality of the mitochondrial isolation procedure was validated as shown in Supplementary Figures 2 and 3.

### Cytokine concentrations

 Supernatant of PBMCs was collected as described above and used for quantifying the cytokines TNF-α, interleukin-6, and interleukin-10 utilizing appropriate human ELISA kits (all BioLegend, San Diego, CA) according to the manufacturers’ instructions. Each cytokine was derived by applying respective calibration standard curves.

For PGC-1α quantification a separate nuclear protein extraction was performed where cells were centrifuged at 4000*g* and the pellet then resuspended in Pre-Extraction Buffer (Abcam, Cambridge, UK) allowing the cells to swell on ice. After vortexing and further centrifugation, the pellet was dissolved in Complete Lysis Buffer (Active Motif, Carlsbad, CA). The lysate was then sonicated to ensure complete lysis and then centrifuged at 13,000*g*. The concentrations of PGC-1α, was measured using a dedicated human ELISA kit (Wuhan EIAab Science Co, Wuhan, China) according to the manufacturers’ instructions.

### Cellular ATP content

The CellTox Green cytotoxicity assay (Promega, Madison, WI) was used to assess the degree of cytotoxicity and remained in all cases less than 15% under our experimental conditions (Supplementary Figure 4). Briefly, cells were seeded in 96-well plates and stimulated as described above. Then CellTox Green reagent was added and incubated for 15 min. Fluorescence was recorded at 520 nm. To assess the cellular ATP content we performed a luciferase-based assay (Cell Titer Glo 2.0 Assay, Promega, Madison, WI) following the manufacturer’s instructions. After assessment of cytotoxicity as described above CellTiter Glow 2.0 reagent was added to the wells and incubated for 10 min. Subsequently the luminescence was recorded.

### Intramitochondrial and extramitochondrial TFAM protein

Western blot was used to assess intramitochondrial and extramitochondrial (= cytonucleoplasmic) TFAM protein levels. Equal amounts of protein were separated on a 4–20% polyacrylamide gradient gel (Bio-Rad, Hercules, CA) and subsequently blotted onto a nitrocellulose membranes (Bio-Rad). The membranes were probed with the primary antibodies against TFAM (1:200; sc-376672, Santa Cruz Biotechnology, Dallas, TX), TNF receptor-associated protein 1 (1:500; Sigma-Aldrich), and beta actin (1:10,000¸ Millipore, Temecula, CA) for five hours at room temperature. After incubation of secondary antibodies (1:10,000; Goat-anti-mouse, IRDye680RD, and 1:10,000; donkey-anti-rabbit, IRDye 800cw, both Li-cor Biosciences, Lincoln, NE) for one hour at room temperature, protein bands were visualized with an Odyssey Scanner (Li-cor Biosciences) and the densitometry was determined using Image J 2.0 software (National Institutes of Health, Bethesda, MD). TRAP1 was used for normalization since its concentration proved to be stable in mitochondria during LPS stimulation (Supplementary Figure 3).

### Expression of TFAM, mitochondrial NADH dehydrogenase subunit 1, and mitochondrial DNA

To assess the gene products by quantitative polymerase chain reaction, total DNA and RNA was extracted from PBMCs using the QIAamp and RNeasy kits respectively, according to the manufacturer’s instructions (QIAGEN, Hilden, Germany). In mRNA samples, the purified RNA was reverse transcribed into complementary DNA using the QuantiTect Reverse Transcription Kit (QIAGEN). Polymerase chain reaction was performed in duplicate using the GoTaq1 qPCR Master Mix (Promega) and specific primers (see Supplementary Figure 5) on a CFX Connect Real-Time System (Bio-Rad Labs). Relative mRNA expression was calculated after normalization using beta actin and ribosomal protein lateral stalk subunit P1 as internal controls using the 2^−∆∆CT^ method^43^.

Mitochondrial DNA copy number was quantified as the ratio of DNA products of mitochondrial NADH dehydrogenase subunit 1 normalized to ribosomal 18S-RNA serving as an internal control using the 2^–∆∆CT^ method^10^.

### Mitochondrial interaction of TFAM with mitochondrial transcription factor 2B

To quantify the mitochondrial protein interactions of TFAM with the Transcription Factor 2B a Proximity Ligation Assay (PLA) was performed^24^. Primary antibodies against TFAM (1:50, sc-376672, Santa Cruz Biotechnology) and mitochondrial Transcription Factor 2B (1:50, 13676, Abcam,) were incubated for 1 h at room temperature. Proximity probes (anti-Mouse Plus; DUO92001 and anti-Goat Minus; DUO92006, both Sigma-Aldrich, each 1:5) were incubated for 1 h at room temperature, S3 splint and S3 backbone oligonucleotides (Biomers.net; Ulm, Germany) were hybridized, ligated and amplified (Supplementary Figure 5). The rolling circle products were visualized with a detection oligonucleotide^44^. Images were submitted to a Cell Profiler pipeline quantifying the proximity ligation assay signals with single cell resolution.

### Statistical analysis

This is the primary analysis of this data. The characteristics of the patients are reported as percentages for categorical variables and as means with SD or medians with interquartile ranges (25th; 75th percentile) as appropriate. Categorical variables were compared using McNemar, or Fisher’s exact tests, as appropriate. Continuous independent variables were compared using the Student’s t­test or the Mann–Whitney test. Continuous dependent variables were compared using the paired samples Student t-test or the Wilcoxon signed-rank test, as appropriate. To explore potential age-related bias, we additionally formed a subgroup only covering controls with an age > 40 years (Supplementary Figure 6). The relationship between mitochondrial TFAM (protein concentration or protein interactions) and the SOFA-score was evaluated using Spearman’s correlation.

A *p-*value of less than 0.05 was considered statistically significant. All CIs were calculated with a coverage of 95%. All analyses were performed using SPSS (version 25, IBM, Chicago, IL, USA). For graphical presentations GraphPad Prism 8 (Graph-Pad, San Diego, CA, USA) was used.

## Supplementary information

Supplementary Information 1.

Supplementary Information 2.

## Data Availability

The complete source data of this manuscript is provided as Supplementary Information.
