# Supplementary material for: Mitochondrial dysfunction in sepsis is associated with diminished intramitochondrial TFAM despite its increased cellular expression
Source: Sci Rep. 2020 Dec 3;10:21029. doi: 10.1038/s41598-020-78195-4 (PMC7713186; doi:10.1038/s41598-020-78195-4)

Supplementary Information

Mitochondrial dysfunction in sepsis is associated with diminished intramitochondrial TFAM abundance despite increased cellular expression

Tim Rahmel, Britta Marko, Hartmuth Nowak, Lars Bergmann, Patrick Thon, Katharina Rump, Sebastian Kreimendahl, Joachim Rassow, Jürgen Peters, Mervyn Singer, Michael Adamzik, Björn Koos

List of contents:

Supplementary Figure 1: Lipopolysaccharide concentration series

Supplementary Figure 2: Representative Western blot of the mitochondrial located voltage-dependent anion-selective channel protein 2 (VDAC2) to evaluate the proper mitochondrial isolation.

Supplementary Figure 3: Representative Western blot of the mitochondrial located TNF Receptor Associated Protein 1 (TRAP1) to evaluate proper mitochondrial isolation.

Supplementary Figure 4: Measure of cellular cytotoxicity

Supplementary Figure 5: Oligonucleotides

Supplementary Figure 6: Age adapted analysis

Supplementary Figure 7: Fully illustrated and unprocessed Western Blots of image excerpts from Figure 1 g.

Supplementary Figure 8: Fully illustrated and unprocessed Western Blots of image excerpts from Figure 1 h.

Supplementary Figure 1: Lipopolysaccharide concentration series

Relative TFAM mRNA expression (quantitative polymerase chain reaction; compared to beta actin) of PBMCs (blue). Cellular ATP was determined using a luciferase-based assay and expressed as relative fluorescent units normalized to 2.25x105 cells per well (red). Cellular cytotoxicity was determined using the CellTox Green assay and expressed as relative cytotoxicity compared lyzed (by 1% Triton X-100) cells (black). Signs with error bars represent mean and SD.

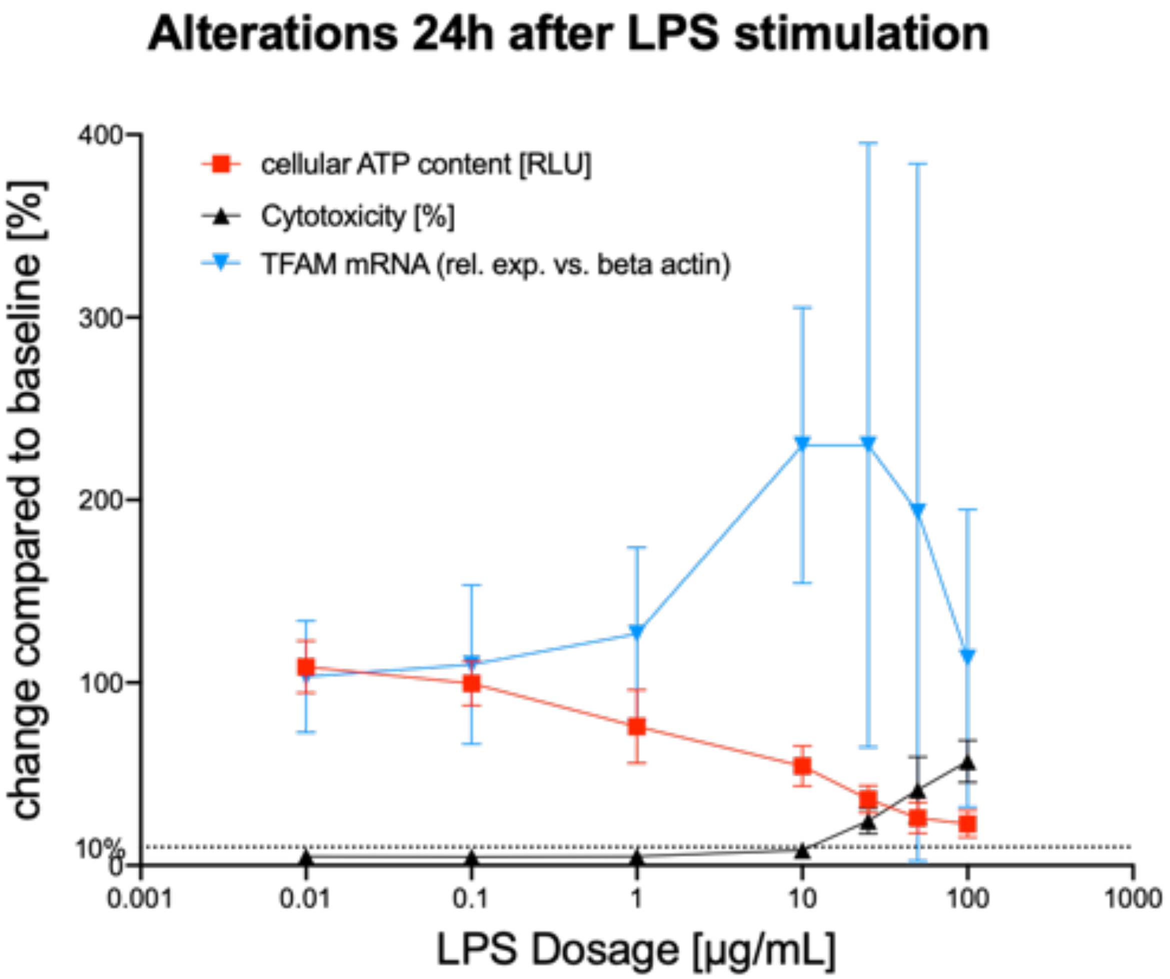

Supplementary Figure 2:
Representative Western blot of the mitochondrial located voltage-dependent anion-selective channel protein 2 (VDAC2) to evaluate the proper mitochondrial isolation. The cytonucleoplasm is free of mitochondria. Red arrow shows specific line of VADAC2 at ~ 30kDa. WCL: whole cell lysate, M: mitochondria; CNP: cytonucleoplasm.

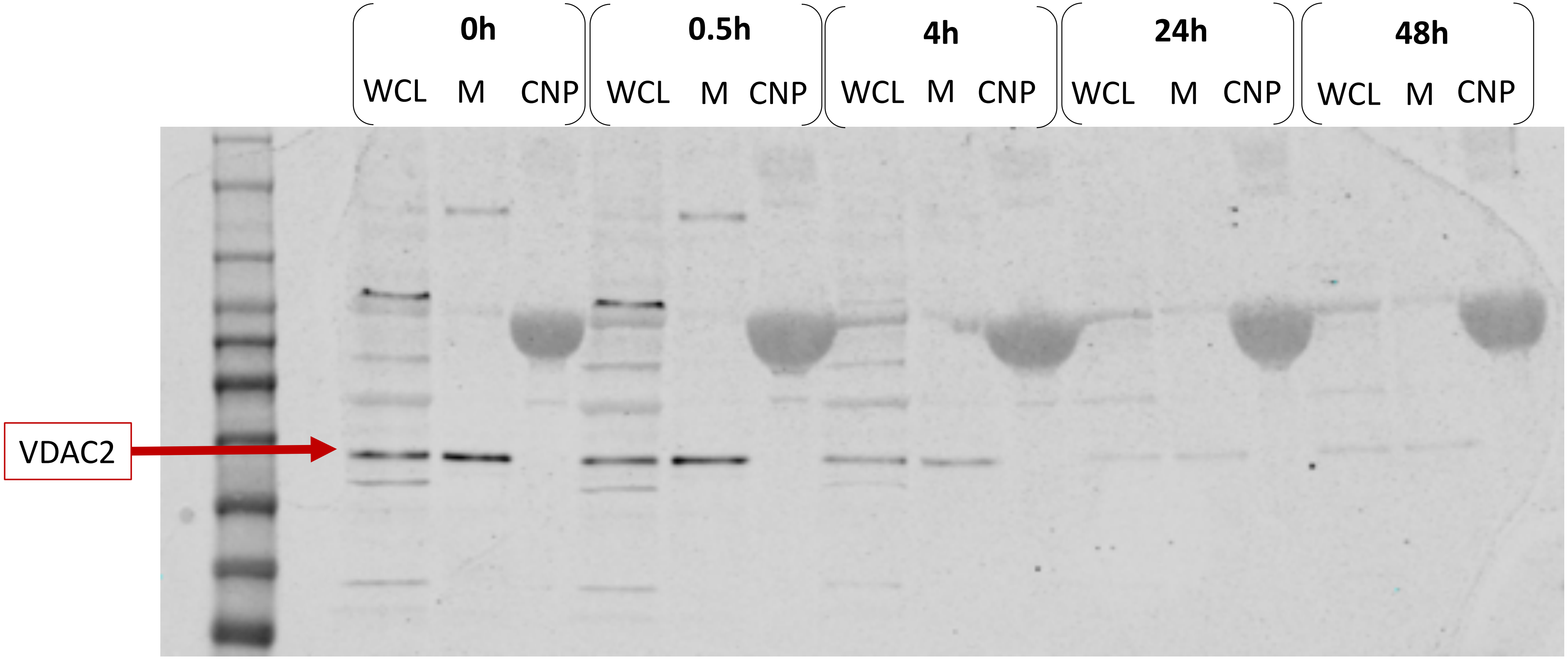

Supplementary Figure 3:

Representative Western blot of the mitochondrial located TNF Receptor Associated Protein 1 (TRAP1) to evaluate proper mitochondrial isolation. The cytonucleoplasm is free of mitochondria. Red arrow shows specific line of TRAP1 at ~ 75kDa. WCL: whole cell lysate, M: mitochondria; CNP: cytonucleoplasm.

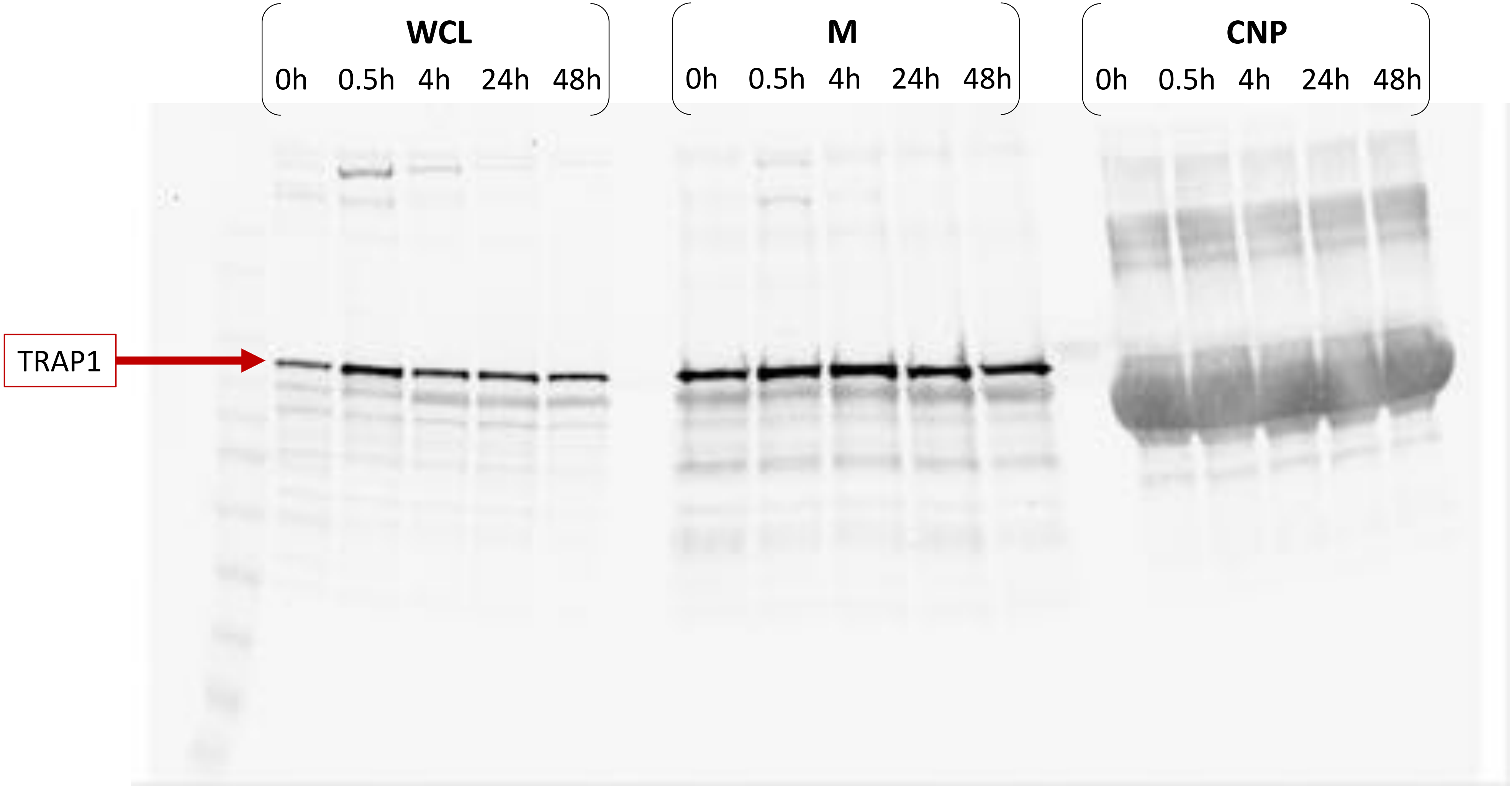

Supplementary Figure 4: Measure of cellular cytotoxicity

To assess the cellular degree of cytotoxicity, we performed the CellTox™ Green Cytotoxicity Assay (Promega, Madison, WI) following the manufacturer's instructions. Briefly, 90 µL of suspended cells (2.5 x 10^6 cells/mL) were seeded with 10µL LPS or 10µL medium into each well. Positive controls were done using 1% Triton X-100. The plates were equilibrated to room temperature and 100 µL reagent was then added. After 10 min incubation at room temperature luminescence measurements were made on a plate reader (Infinite M200PRO).

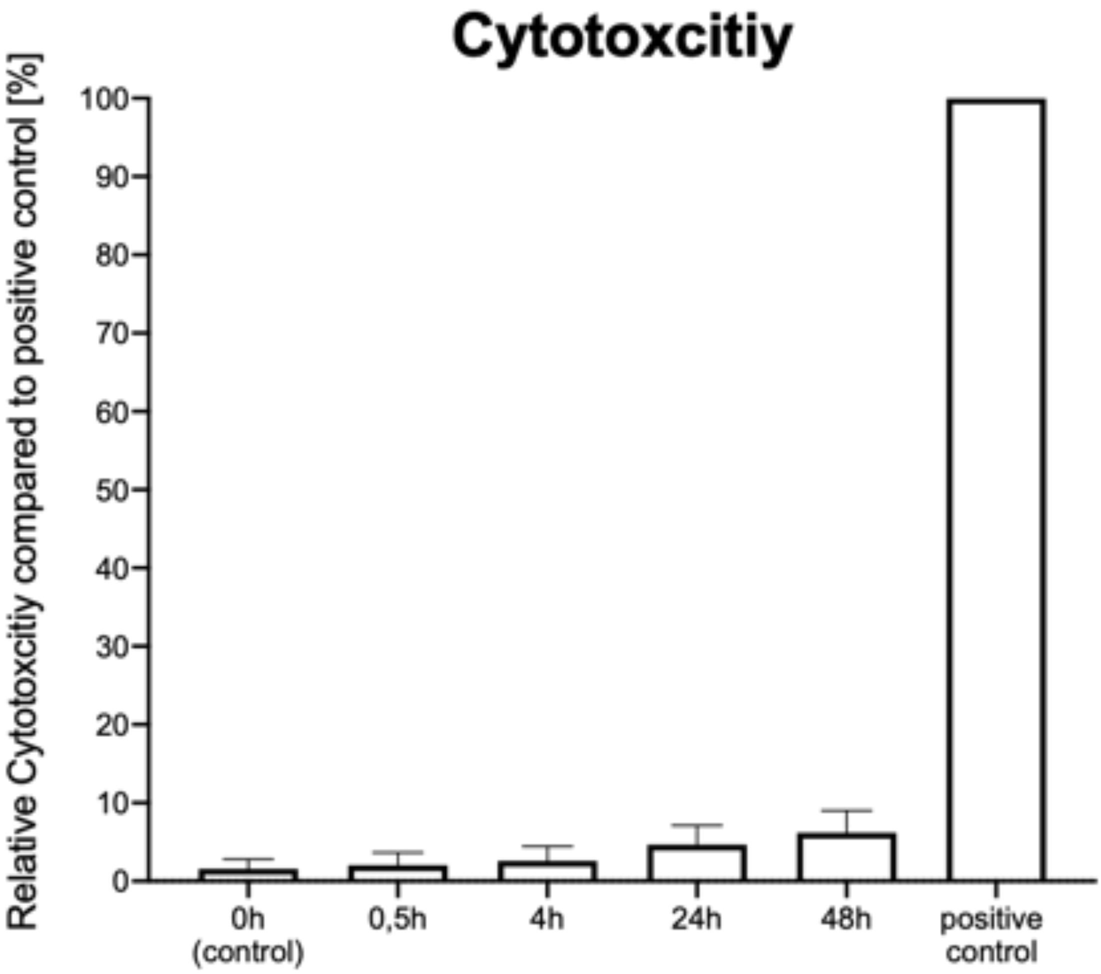

Supplementary Figure 5: Oligonucleotides

Oligonucleotide pairs used for quantitative polymerase chain reaction

Oligonucleotide name	Sequence
**mRNA (c-DNA) targets**	
PGC-1α forward	5´-TGAACTGAGGGACAGTGATTTC-3´
PGC-1α reverse	5´-CCCAAGGGTAGCTCAGTTTATC-3´
TFAM forward	5´-CTCAGAACCCAGATGCAAA-3´
TFAM reverse	5´-GCCACTCCGCCCTATAA-3´
Beta actin forward	5´-CATGTACGTTGCTATCCAGGC-3´
Beta actin reverse	5´-CTCCTTAATGTCACGCACGAT-3´
Mitochondrial NADH dehydrogenase subunit 1 forward	5´-TGGGTACAATGAGGAGTAGG-3´
Mitochondrial NADH dehydrogenase subunit 1 reverse	5´-GGAGTAATCCAGGTCGGT-3´
Ribosomal protein lateral stalk subunit P1 forward	5´-AGCCGGTGTAAATGTTGAGCCT-3´
Ribosomal protein lateral stalk subunit P1 reverse	5´-TCTTTGCTTCCACTTTCTTCTCCT-3´
**DNA targets**	
Mitochondrial NADH dehydrogenase subunit 1 forward	5´-CACCCAAGAACAGGGTTTGT-3´
Mitochondrial NADH dehydrogenase subunit 1 reverse	5´-TGGCCATGGGTATGTTGTTAA-3´
18SrRNA forward	5´-TAGAGGGACAAGTGGCGTTC-3´
18SrRNA reverse	5´-CGCTGAGCCAGTCAGTGT-3´

Sequences of oligonucleotides used in the Proximity Ligation Assay

Oligonucleotide	Sequence
S3 Backbone	5’-phos-ctattagcgtccagtgaatgcgagtccgtctaagagagtagtacagca gccgtcaagagtgtcta-3’
S3 Splint	5’-phos-gttctgtcatatttaagcgtcttaa-3’
Compaction Oligonucleotide	5’-agagagtagtacagcagccgtaaaagagagtagtacagcagccgtUUU-3’
Detection Oligonucleotide	5’-Atto550-cagtgaatgcgagtccgtct-3’

Supplementary Figure 6: Age adapted analysis

Results of PBMCs from septic patients (n=10; blue bars) sampled within 24 hours after diagnosis of sepsis compared to healthy controls (n=9; grey bars). **Upper panel:** Concentration of selected cytokines in PBMC supernatants. **a** TNF-α, **b** Interleukin-6, and **c** Interleukin-10. **d** Peroxisome proliferator-activated receptor gamma coactivator 1-alpha (PGC-1α) level (ELISA of nuclear protein extracts). **e** Relative TFAM mRNA expression (quantitative polymerase chain reaction; compared to beta actin) of PBMCs; AU: arbitrary units.**f**,**g** Relative TFAM protein expression in cytonucleoplasm normalized to beta actin (**f**) and relative mitochondrial TFAM protein amount normalized to TNF receptor-associated protein 1 (**g**). Each circle represents an individual volunteer / patient; columns with error bars represent mean and SD. There were no missing data. P values were determined using the Mann-Whitney test; *p<0.05, **p < 0.01, ***p < 0.001. All exact values are presented in the Source Data file.

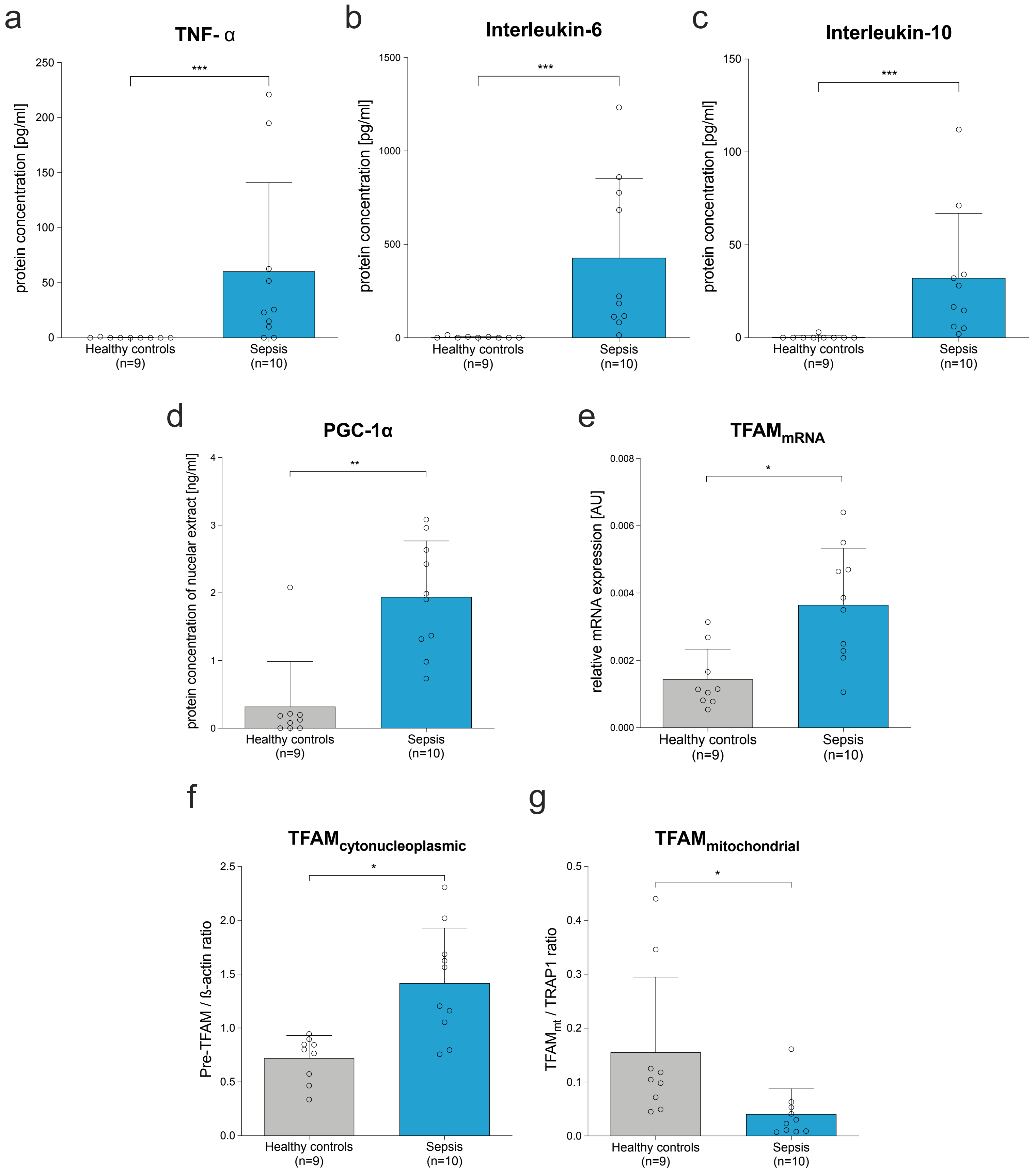

Supplementary Figure 7:

Fully illustrated and unprocessed Western Blots of image excerpts from Figure 1 g. **a** TFAM of cytonucleoplasm. Red arrow shows specific line of cytonucleoplasmic TFAM at ~29kDa. **b** ß-actin of cytonucleoplasm. Red arrow shows specific line of cytonucleoplasmic ß-actin ~42kDa.

a

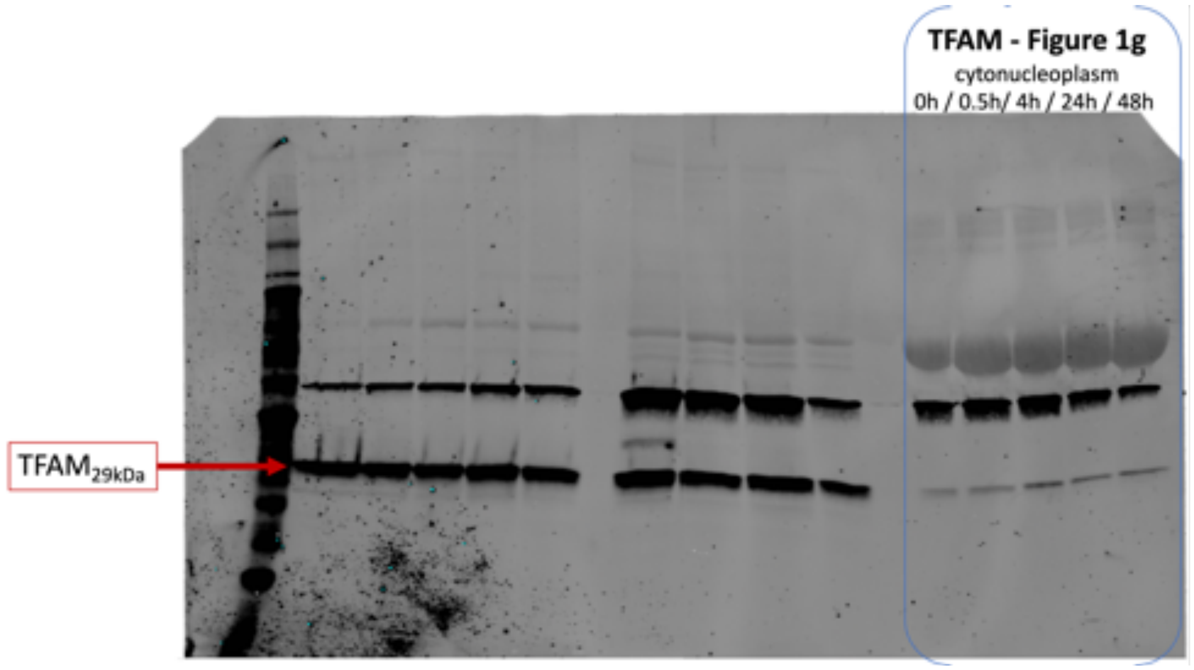

b

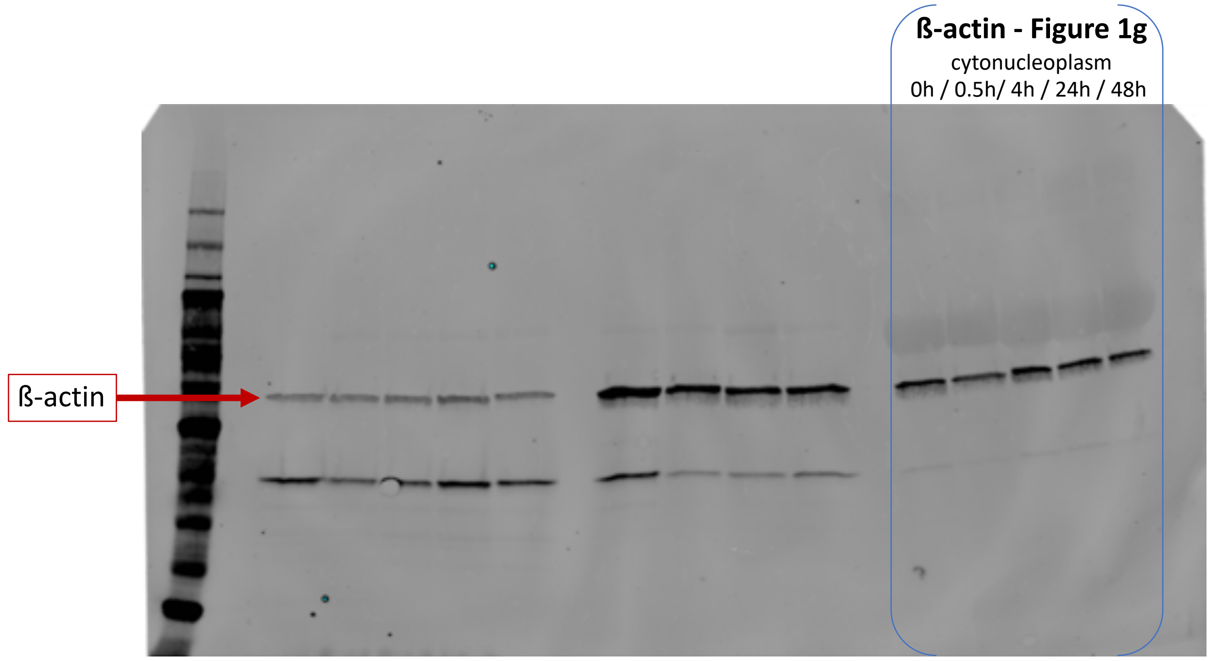

Supplementary Figure 8:

Fully illustrated and unprocessed Western Blots of image excerpts from Figure 1 h. **a** TFAM of mitochondria. Red arrow shows specific line of mitochondrial TFAM at ~24kDa. **b** TRAP1 of mitochondria. Red arrow shows specific line of TRAP1 at ~75kDa.

a

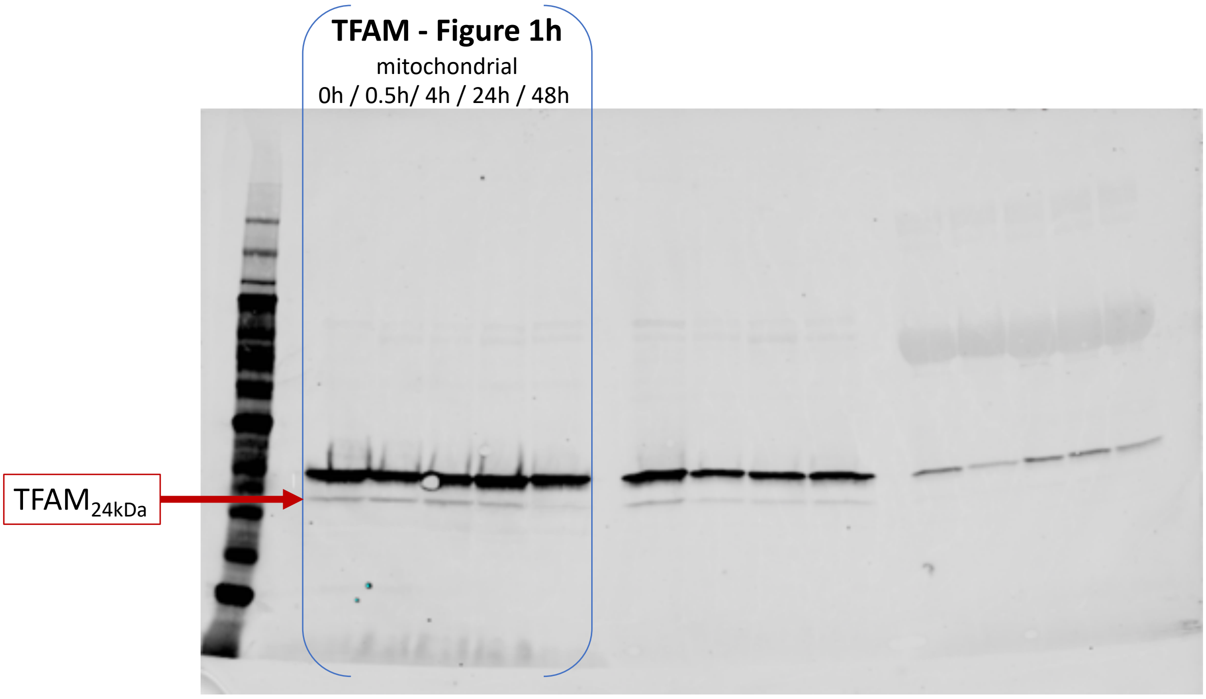

b

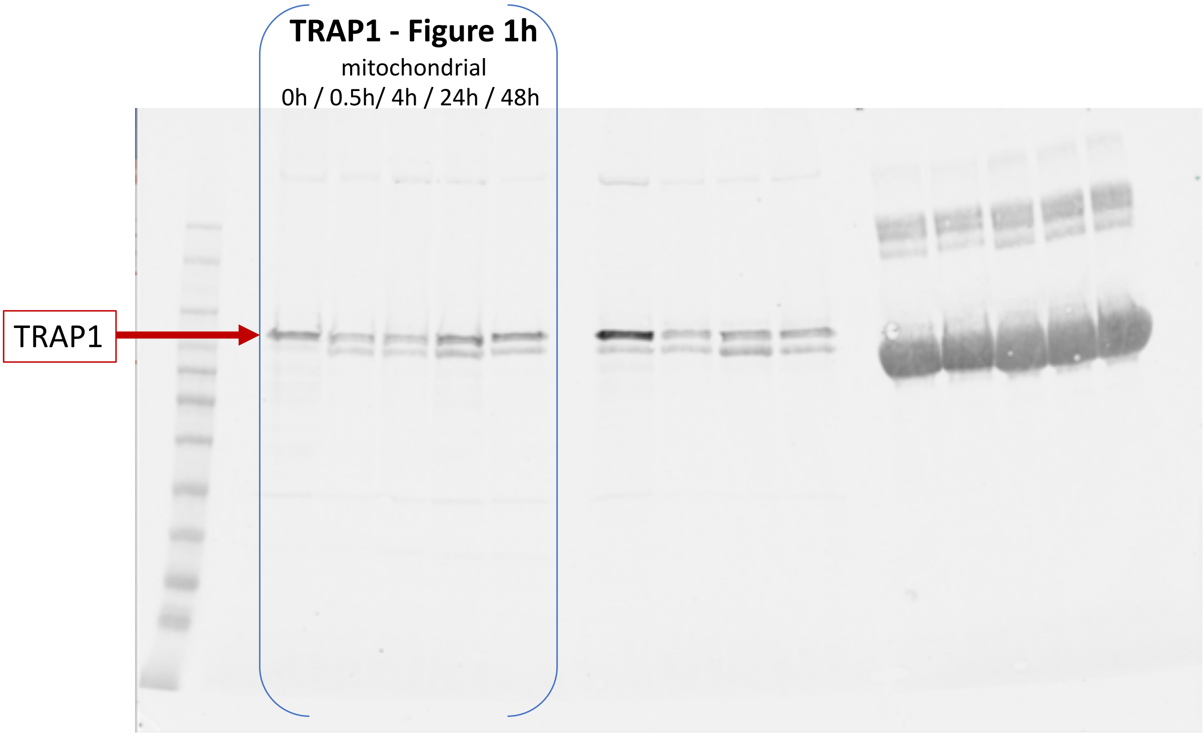

Supplement: Supplementary file 1 — Supplementary Information 1. [file 41598_2020_78195_MOESM1_ESM.pdf]
